# Rare Driver Mutations in Advanced, Oncogene-Addicted Non-Small Cell Lung Cancer: A North Italian, Real-World, Registry Experience

**DOI:** 10.3390/diagnostics14101024

**Published:** 2024-05-15

**Authors:** Kalliopi Andrikou, Paola Ulivi, Elisabetta Petracci, Irene Azzali, Federica Bertolini, Giulia Alberti, Stefania Bettelli, Daniele Calistri, Elisa Chiadini, Laura Capelli, Paola Cravero, Giorgia Guaitoli, Francesca Zanelli, Marco Angelo Burgio, Maria Pagano, Alberto Verlicchi, Enrica Martinelli, Katia Di Emidio, Massimo Dominici, Carmine Pinto, Angelo Delmonte

**Affiliations:** 1 Medical Oncology, IRCCS Istituto Romagnolo per lo Studio dei Tumori “Dino Amadori” (IRST), 47014 Meldola, Italy; kalliopi.andrikou@irst.emr.it (K.A.); paola.cravero@irst.emr.it (P.C.); marco.burgio@irst.emr.it (M.A.B.); alberto.verlicchi@irst.emr.it (A.V.); angelo.delmonte@irst.emr.it (A.D.); 2 Bioscience Laboratory, IRCCS Istituto Romagnolo per lo Studio dei Tumori “Dino Amadori” (IRST), 47014 Meldola, Italy; daniele.calistri@irst.emr.it (D.C.); elisa.chiadini@irst.emr.it (E.C.); laura.capelli@irst.emr.it (L.C.); 3 Unit of Biostatistics and Clinical Trials, IRCCS Istituto Romagnolo per lo Studio dei Tumori (IRST) “Dino Amadori”, 47014 Meldola, Italy; elisabetta.petracci@irst.emr.it (E.P.); irene.azzali@irst.emr.it (I.A.); 4 Oncology Department, Modena University Hospital, 41125 Modena, Italy; bertolini.federica@policlinico.mo.it (F.B.); giorgia.guaitoli@unimore.it (G.G.); enrichelli14@gmail.com (E.M.); diemidio.katia@aou.mo.it (K.D.E.); massimo.dominici@unimore.it (M.D.); 5 Medical Oncology, IRCCS Arcispedale Santa Maria Nuova, 42123 Reggio Emilia, Italy; giulia.alberti@ausl.re.it (G.A.); zanelli.francesca@ausl.re.it (F.Z.); maria.pagano@ausl.re.it (M.P.); carmine.pinto@ausl.re.it (C.P.); 6 Biomolecular Pathology Unit, Azienda Ospedaliera Unica di Modena, 41125 Modena, Italy; bettelli.stefania@aou.mo.it

**Keywords:** NSCLC, oncogene addiction, inflammatory indexes

## Abstract

The real-world, retrospective, NEROnE registry investigated the impact of next-generation sequencing (NGS) in advanced non-small-cell lung cancer (NSCLC) patients (pts) at three oncology units in the north of Italy between January 2020 and December 2022. We focused on the clinical characterization and outcomes of NSCLC with rare molecular alterations: *EGFR* exon 20 insertion, non-activating *EGFR* mutations, *BRAF* V600E and non-V600, *ROS1* and *RET* rearrangements, *MET*, ErbB2, and *FGFR* mutations. Overall, these represented 6.4% (62/970) of the pts analysed with NGS in the daily practice. The most heavily represented rare alterations were ROS1 rearrangement (15 pts—24%) and MET exon 14 skipping mutation (11 pts—18%). No associations were found with the demographic and clinical features. Forty-nine pts received targeted therapies, of which 38.8% were first- and 9.8% were second-line. The remaining pts received chemotherapy and/or immunotherapy. In terms of the clinical outcomes, although not statistically significant, a tendency toward shorter OS was seen when therapies other than specific targeted therapies were used (HR: 1.84, 95% CI: 0.79–4.33, *p* = 0.158). The pts with co-mutations (19.4%) seemed to receive an advantage from the front-line chemotherapy-based regimen. Finally, an NLR score (a well-known inflammatory index) ≥ 4 seemed to be related to shorter OS among the pts treated with immunotherapy alone or in combination with chemotherapy (HR: 2.83, 95% CI: 1.08–7.40, *p* = 0.033). Prospective evaluations need to be performed to clarify whether these indexes may help to identify patients with oncogene-addicted NSCLC who could benefit from immunotherapy.

## 1. Introduction

Lung cancer is one of the most frequent types of cancer in developed countries, accounting, in the USA, for almost a quarter of cancer-related deaths and being the leading and second-leading cause of cancer in Europe for men and women, respectively [1]. Among lung cancers, non-small-cell lung cancer (NSCLC) represents more than 85% of all cases, with adenocarcinoma being the most heavily represented histological subtype [2]. Although they are still crucial, currently, histologic features in NSCLC are not enough to define the correct therapeutic strategy. In fact, the discovery of epidermal growth factor receptor (*EGFR*)-activating mutations and anaplastic lymphoma kinase (*ALK*) gene rearrangements and their specific treatments [3,4] has changed the therapeutic landscape, given their ability to modify outcomes for many patients with such alterations. Moreover, several retrospective multicentric evaluations have shown that the outcomes of patients with oncogene-addicted NSCLC are significantly improved when therapies are given according to the target [5,6]. Furthermore, data from Surveillance, Epidemiology, and End Results (SEER) have highlighted improvements in incidence-based mortality greater than in previous periods since 2013, in men, and since 2014, in women, considering that since 2013, the use of EGFR inhibitors has been approved as a first-line treatment for advanced NSCLC [7]. For these reasons it has become mandatory to define the molecular hallmarks of each NSCLC in order to use the most appropriate and active treatment from the beginning. At present, it is mandatory to define the molecular status of of ten oncogenes from the point of diagnosis, preferentially through simultaneous next-generation sequencing (NGS): mutations of *EGFR* (activation and insertion of exon 20), *KRAS*, *BRAF*, *MET*, *HER2*, and *FGFR* and rearrangements of *ALK*, *ROS1*, *RET*, and *NTRK* [8,9]. In fact, for all of these molecular alterations, in first or in further lines of treatment, a specific targeted treatment is available in routine practice or in the advanced stage of clinical development: osimertinib for EGFR-activating mutations [10]; amivantamab for the insertion of exon 20 of EGFR [11]; sotorasib and adagrasib for KRAS G12C mutations [12]; dabrafenib plus trametinib for BRAF V600 mutations [13]; capmatinib and tepotinib for MET skipping mutations [14]; trastuzumab and deruxtecan for HER2 mutation [15]; AZD4547 for FGFR alterations [16]; alectinib, brigatinib, and lorlatinib for ALK rearrangements [17]; crizotinib, entrectinib, and repotrectinib for ROS1 rearrangements [18]; selpercatinib for RET rearrangement [19]; and entrectinib and larotrectinib for NTRK rearrangement [20]. Finally, the expression of PD-L1 needs to be obtained by immunohistochemistry in order to clarify responsiveness to immunotherapy [21].

The NEROnE study is an observational, retrospective registry aiming to define the real-world impact of molecular testing on NSCLC characterization and outcomes for patients with at least one of the ten molecular targets for which a specific therapy is available, approved by national regulatory authorities or in clinical trials available in Italy in the period of observation. In the NEROnE study, data were obtained from the routine clinical practice of three oncologic units located in the Emilia Romagna Region in the north of Italy: the patients involved were molecularly defined by NGS analyses according to the local standard of diagnosis and cure. No supplementary molecular characterizations, other than those approved by the Italian regulatory authorities, were performed. In this paper, we focus on the rarest molecular subpopulations among the mandatory ten, describing their clinical features and outcomes according to the therapies approved in Italy for each subtype following the initial diagnosis. 

## 2. Materials and Methods

### 2.1. Study Design and Population

NEROnE is a retrospective, real-world, observational registry of patients with advanced NSCLC carrying at least one driver mutation in 10 genes for which, at present, targeted therapies are available, approved by the government, or in clinical trials: EGFR exon 20 insertion, non-activating EGFR mutations, BRAF V600E and non-V600, ROS1, RET, and NTRK rearrangements, and MET, ErbB2, and FGFR mutations. All consecutive patients tested with NGS as per clinical practice between January 2020 and December 2022, presenting driver mutations, and starting a first-line treatment at three centres in Northern Italy were included in this study. The three oncology units involved were: AUSL-IRCCS Arcispedale Santa Maria Nuova, in Reggio Emilia, and Modena University Hospital and IRCCS Istituto Romagnolo per lo Studio dei Tumori “Dino Amadori” (IRST), in Meldola (FC). Considering NGS procedures, samples from Modena and Reggio Emilia were analysed at Modena University Hospital Molecular Pathology Laboratory using Myriapod IL- 56G, Cancer Panel DNA, Cancer Panel RNA, Oncomine DX Target Test Assay (Diatech Pharmacogenetics, Jesi, Italia), and Oncomine Focus (ThermoFisher Scientific, Waltham, MA, USA). Samples from IRST Meldola were studied at its Molecular Diagnostic Unit with Myriapod NGS cancer panel DNA and RNA, Oncomine Focus Assay, and Oncomine Comprehensive Assay v3, (Thermo Fisher Scientific, Waltham, MA, USA). In the present substudy, only those with molecular alterations with an incidence of less than 2.5% were considered, and defined as infrequent mutations. Clinical and laboratory information were obtained from medical chart review. 

### 2.2. Immunoscores Definition

The white blood cell count and differential counts, evaluated at the baseline, were used to determine neutrophil-to-lymphocyte ratio (NLR) [22], platelet-to-lymphocyte ratio (PLR) [23], advanced lung cancer inflammation index (ALI) [24], and the systemic immune inflammatory (SII) index [25]. NLR was computed as the ratio of the absolute neutrophil count to the absolute lymphocyte count, PLR as the ratio of absolute platelet count to the absolute lymphocyte count, ALI as BMIxALB/NLR, where BMI = body mass index and ALB = serum albumin g/dL, and SII as platelet count × neutrophil count/lymphocyte count.

### 2.3. Statistical Analysis

Data were summarised by the median, first (IQ), and third (IIIQ) quartiles, by minimum and maximum values for continuous variables, and by means of absolute frequencies and percentages for categorical variables.

Comparisons between categorical variables were performed using the Pearson’s χ^2^ test of the Fisher exact test, as appropriate, whereas those between categorical and continuous variables were performed through the Wilcoxon signed-rank sum test or the Kruskal–Wallis test, as appropriate.

The inflammatory indexes were reported as log-transformed continuous variables and as categorical variables using the median as a cut-off value.

Progression-free survival (PFS) was defined as the time in months between the start of first-line treatment and the date of disease progression, death from any cause, or last follow-up, whichever occurred first. Overall survival (OS) was defined as the time in months between the start of first-line treatment and the date of death from any cause or last follow-up, whichever occurred first. These outcomes were analysed using the Kaplan–Meier method, the log-rank test for group comparisons, and the Cox proportional hazards model. Results were reported as median and in terms of hazard ratios (HRs) and corresponding 95% confidence intervals (CI)s. The median follow-up time was computed using the reverse Kaplan–Meier method. 

All analyses were carried out with STATA 15.0 (College Station, TX, USA). Results were considered statistically significant if the two-sided *p*-values were <0.05.

## 3. Results

### 3.1. Molecular and Clinical Features

**Figure 1:**
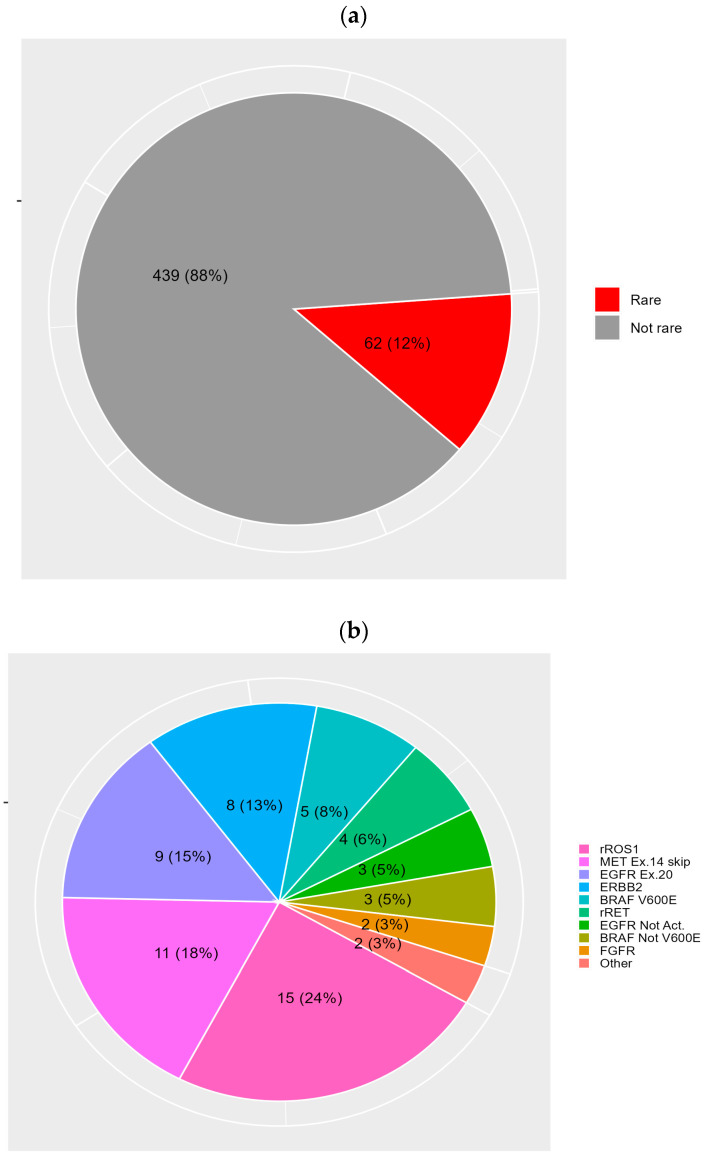
(**a**) Prevalence of rarest molecular alterations among the whole oncogene-addicted population of the NEROnE study: the red slice represents patients with the rarest mutations, accounting for 12% of the population; (**b**) prevalence distribution of the type of rare mutation considered in the present study.

Nine-hundred seventy patients with a new diagnosis of NSCLC underwent NGS, and 501 patients with at least one druggable oncogene mutation were identified, representing the overall population of the NEROnE study. Among these 501 patients, 62 (12.4%) had rare driver mutations: nine (1.8%) showed *EGFR* exon 20 mutations, three (0.6%) showed non-activating *EGFR* mutations, five (1%) showed *BRAF* V600E mutations, three (0.6%) showed *BRAF* non-V600 mutations, 15 (3%) showed *ROS1* and four (0.8%) showed *RET* rearrangements, 11 (2.2%) showed *MET* exon 14 skipping mutations, eight (1.6%) showed ErbB2 mutations, two (0.4%) showed *FGFR* mutations, and two (0.4%) showed other molecular alterations (Figure 1). No *NTRK* rearrangements were found. If we consider the total of 970 patients analysed with NGS, those with a rare mutation represent 6.4%.

The patients had a median age of 69.2 years (35.2–87.00); 27 (43.5%) were male and 35 (56.5%) were female; 23 (37.1%) were never-smokers, 28 (45.2%) were previous smokers, and 11 were current smokers (17.7%). Sixty (96.8%) patients had adenocarcinoma and two (3.2%) had squamous cell carcinoma; the expression of PD-L1 was <1% in eight (13.1%) patients, between 1 and 49% in 35 (57.4%), and ≥50% in 18 (29.5%); and the PD-L1 status was missing in one patient. The main patients characteristics are reported in Table 1.

No associations were detected between the molecular features and specific clinical features, as shown in Table 2.

### 3.2. Treatments and Clinical Outcomes

The median follow-up time was 25.1 months (95% CI, 18.4–30.2). All the patients received at least one line of treatment. Overall, the median progression-free survival (PFS) and median overall survival (OS) were 5.0 months (95% CI, 3.1–13.3) and 20.7 months (95% CI, 8.2—not reached (NR)), respectively. Twenty-four (38.8%) patients received targeted agents as first-line therapy (group 1), including crizotinib [13], afatinib [1], dabrafenib and trametinib [4], and capmatinib [2], and four patients were enrolled in clinical trials. Thirty-eight (61.2%) patients received non-targeted therapies (group 2): among them, 20 received chemo–immunotherapy, 10 received immunotherapy alone, five received platinum-based chemotherapy doublets, and three had single-agent chemotherapy.

**Figure 2:**
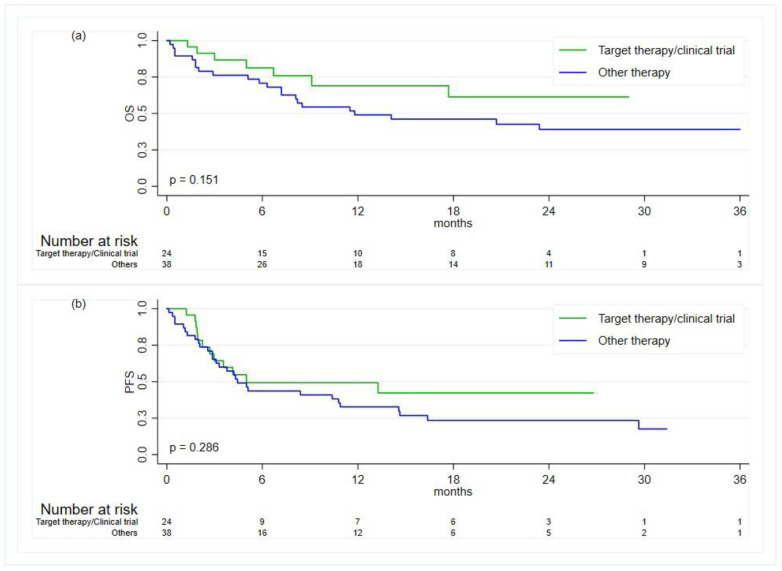
Kaplan–Meier curves for the comparison of the clinical outcomes between patients treated with targeted therapies or in clinical trials and patients treated with chemotherapy and/or immunotherapy: (**a**) overall survival (OS); (**b**) progression-free survival (PFS).

The median duration of treatment in group 1 was 3.6 months (IQ-IIIQ: 1.6–15.8), and it was 4.3 months (IQ-IIIQ: 2.0–14.6) in group 2; the *p*-value = 0.851. The median OS rates at 12 and 24 months were 69% (95% CI, 42–85%) and 61% (95% CI, 34–80%) in group 1 and 49% (95% CI, 32–64%) and 39% (95% CI, 23–55%) in group 2. The median PFS rates at 12 and 24 months were 49% (95% CI, 27–68%) and 42% (95% CI, 20–63%) in group 1 and 33% (95% CI, 18–48%) and 23% (95% CI, 11–38%) in group 2. No statistically significant differences were observed between the treatment group with respect to the OS (*p*-value = 0.151) and PFS (*p*-value = 0.286) (Figure 2). Among the patients in group 2, the median OS rates were 23.4 months (95% CI: 7.2–not reached (NR)), 2 months (95% CI: 0.4–NR), and 6.3 months (95% CI: 0.2–14.1) for the patients treated with chemo–immunotherapy, with immunotherapy alone, and with chemotherapy alone, respectively. The corresponding estimates for PFS were 8.4 months (95% CI: 3.3–29.6), 2 months (95% CI: 0.4–16.4), and 2.6 (95% CI: 0.13–10.39), respectively.

Overall, 21 (33.9%) patients received a second line of therapy, of whom five were in group 1 and 16 were in group 2 (*p*-value = 0.534). Among the five patients in group 1, two (40%) received targeted therapies; among the 16 in group 2, six (37.5%) received targeted therapies or participated to clinical trials. This latter group represents the 9.8% of the population analysed.

### 3.3. Patients with Co-Mutations

Twelve out of 62 patients (19.4%) presented with a co-mutation: five out of 62 (8%) presented a *ROS1* rearrangement synchronous with *EGFR* rare mutations (exon 20 insertion or inactivating mutation), a *KRAS* G12C mutation, or other non-target alterations; one out of 62 (1.6%) presented a *BRAF* V600E mutation with a PIK3CA mutation; one out of 62 (1.6%) presented a non-V600 *BRAF* mutation and a PIK3CA mutation; three out of 62 (4.8%) presented a *MET* exon 14 skipping mutation with *RET* rearrangement, with an erbB2 mutation, or with other non-target alterations; and one out of 62 (1.6%) presented with two synchronous mutations of *FGFR* genes. These patients showed similar characteristics to those with a single driver mutation, with no statistically significant differences (results not shown).

The patients with co-mutations had a median OS of 17.7 months (95% CI, 1.9–NR) versus 20.7 months (95% CI, 8.1–NR) for the patients with only one oncogene alteration, with *p*-value = 0.907. The corresponding medians for PFS were 4.1 months (95% CI, 1.8–10.9) and 5.1 months (95% CI, 3.1–14.6), with *p*-value = 0.191, respectively. Among the patients with co-mutations, five (41.7%) received first-line targeted therapies or participated in clinical trials, while seven (58.3%) received chemotherapy alone or combined with immunotherapy, with *p*-value = 0.815. The corresponding median durations of treatment were 2.2 months (IQ-IIIQ: 1.8–3.2) and 10.8 months (IQ-IIIQ: 2.9–29.6), with *p*-value = 0.123, respectively. The median OS for the patients with co-mutations and treated with targeted therapies or in clinical trials was equal to 17.7 months (95% CI, 1.9–NR) and NR (95% CI, 0.5–NR) for those receiving other treatments, *p*-value = 0.459. The corresponding median PFSs were 2.2 months (1.8–NR) and 10.8 months (0.5–29.6), with *p*-value = 0.052.

### 3.4. Association with Inflammatory Indexes

**Figure 3:**
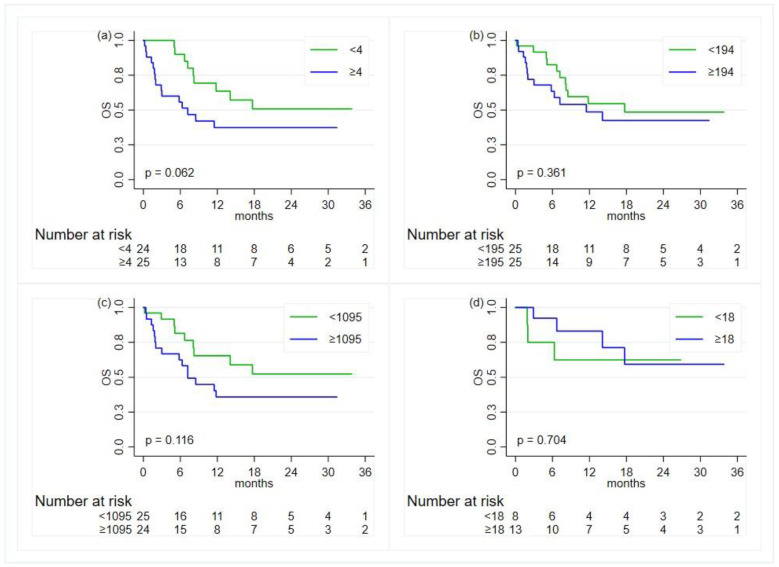
Kaplan–Meier curves for the comparison, in the overall population, of the overall survival (OS) between groups defined by the status of the inflammatory indexes—except for ALI, the cut-offs are based on the median value: (**a**) neutrophil-to-lymphocyte ratio; (**b**) platelet-to-lymphocyte ratio; (**c**) systemic immune-inflammation index; (**d**) advanced lung cancer inflammation index.

Overall, on the univariate analysis, there was some evidence of a worse OS for patients with a baseline NLR index greater than or equal to 4 compared to patients with lower values (HR 2.3; 95% CI, 0.94–4.93, *p*-value = 0.069) (Table 3 and Figure 3). However, when adjusting for age at diagnosis and ECOG performance status (PS), the NLR was not found to be associated with OS (HR = 1.92, 95% CI, 0.82–4.45, *p*-value = 0.131).

**Figure 4:**
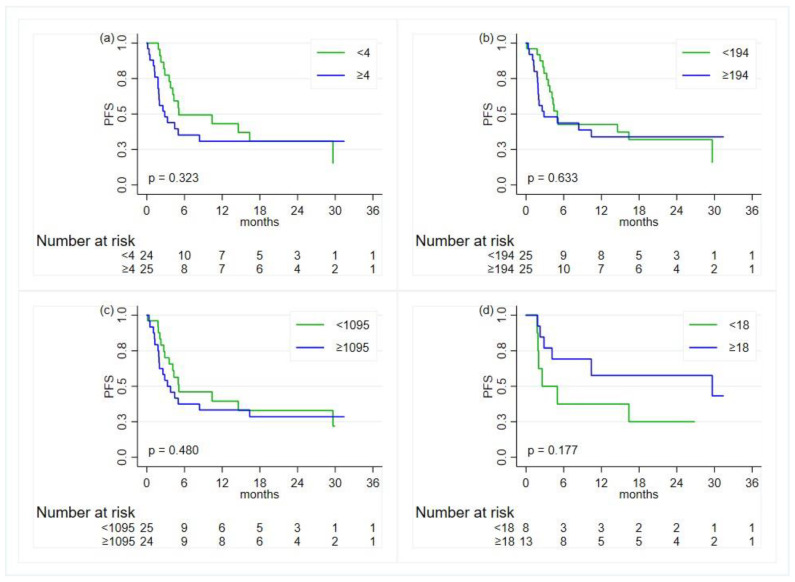
Kaplan–Meier curves for the comparison, in the overall population, of the progression-free survival (PFS) between groups defined by the status of the inflammatory indexes—except for the ALI, the cut-offs are based on the median value: (**a**) neutrophil-to-lymphocyte ratio; (**b**) platelet-to-lymphocyte ratio; (**c**) systemic immune-inflammation index; (**d**) advanced lung cancer inflammation index.

With regards to the PFS, no associations were found (Table 3 and Figure 4).

**Figure 5:**
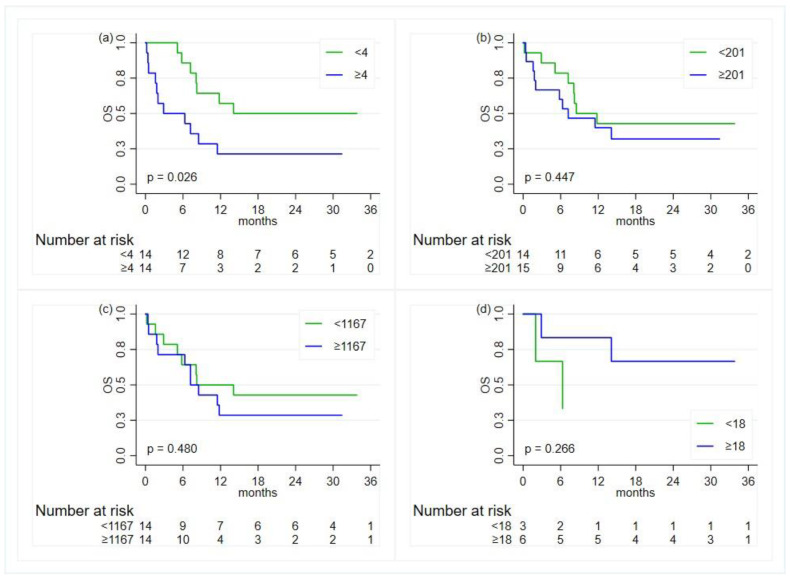
Kaplan–Meier curves for the comparison, in the population treated with chemotherapy and/or immunotherapy, of the overall survival (OS) between groups defined by the status of the inflammatory indexes—except for ALI, the cut-offs are based on the median value: (**a**) neutrophil-to-lymphocyte ratio; (**b**) platelet-to-lymphocyte ratio; (**c**) systemic immune-inflammation index; (**d**) advanced lung cancer inflammation index.

Looking at the association between the inflammatory indexes and the time-to-event outcomes within the groups of treatment, a statistically significant association with OS (HR 2.8, 95% CI, 1.08–7.40, *p*-value = 0.033) was observed for the NLR in the subgroup of patients treated with chemotherapy, immunotherapy, or their combination (Figure 5). Adjusting for age at diagnosis and ECOG PS, a HR of 2.63 (95% CI, 0.96–7.18, *p*-value = 0.060) was found.

**Figure 6:**
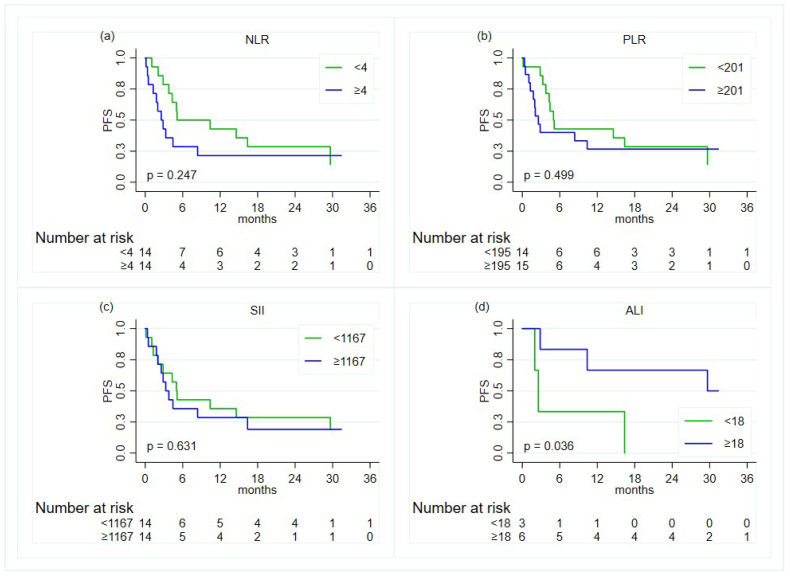
Kaplan–Meier curves for the comparison, in the population treated with chemotherapy and/or immunotherapy, of the progression-free survival (PFS) between groups defined by the status of the inflammatory indexes—except for ALI, the cut-offs are based on the median value: (**a**) neutrophil-to-lymphocyte ratio; (**b**) platelet-to-lymphocyte ratio; (**c**) systemic immune-inflammation index; (**d**) advanced lung cancer inflammation index.

No other significant associations were observed with the OS. Concerning the PFS, there was some evidence of an association with the ALI index (HR 0.18, 95% CI, 0.03–1.09, *p*-value = 0.061) (Figure 6). However, in this subgroup, ALI data were available only for nine patients (six failures).

Among the patients treated with target therapy or within a clinical trial, no associations were found.

## 4. Discussion

The data from the whole oncogene-addicted population of the NEROnE study, a real-world, retrospective data collection from three oncologic units in the north of Italy, were previously shown [26]. In the present work, we describe the patients with the rarest molecular, driver alterations among the pivotal ten analysed in the study. Considering that data for the activity of new drugs for NSCLC subpopulations arise from controlled clinical trials, real-world information is required in order to verify results from everyday clinical practice, especially for the less frequent alterations. 

In our case series, cases of NSCLC with the rarest driver alterations encompassed6% of the overall population who underwent NGS. Each subgroup shows a prevalence lower than in the literature, where, in fact, *EGFR* exon 20 mutations represent2.5% of the analysed patients [27], *BRAF* mutations represent 3% [28], *RET* rearrangements represent 1–2% [29], *MET* exon 14 skipping mutations represent 2.7% [30], and *FGFR* mutations/rearrangements represent 0.1–3% [31]. Only the *ROS1*-rearranged and the erbB2-mutated patients had incidences similar to that of in the literature, of 2% and 1%, respectively [6,16]. The patients with these alterations did not show associations with specific clinical features in our data. In the literature, although not all subgroups show well defined clinical features, it is possible to highlight some specificities: *EGFR* exon 20 mutants are more frequent in non-smoker patients [27], while the majority of BRAF-mutated patients are current or former smokers [28]; ROS1- and RET-rearranged patients are generally younger than 60 years and have limited smoking history [29,30,31,32]; and MET mutations are more frequent in older patients [30]. In our dataset, as in the literature, there are no clear correlations with PD-L1 expression [33].

From a therapeutic point of view, at present, in Italy, only a limited number of therapeutic options for these oncogene-addicted populations is available. First-line targeted therapies for BRAF-V600-mutated and ROS1-rearranged NSCLC and second-line therapies for the *EGFR* exon 20 mutation, *MET* exon 14 skipping mutation, and RET rearranged NSCLC are approved by regulatory autorities; treatments for erbB2 and FGFR are still under investigation. Only 48.6% of our patients had received targeted therapies or participated in dedicated clinical trials, of which 38.8% were first-line and 9.8% were second-line, respectively. Although there are no statistical differences in terms of OS and PFS between patients treated with targeted therapies and chemo-based regimens in the first line, there is a trend in terms of improvement in the median rates of OS and PFS at 12 and 24 months for those receiving targeted therapies. These results are consistent with the literature, in which a generic population with oncogene-addicted tumours had better outcomes when treated with specific drugs [5,6]. In our population, these results are not statistically significant, probably due to the small sample size and its heterogeneity, but also because not all patients can receive the most appropriate therapy as an initial option.

As in the historical data [34], in our study population, there was an incidence of NSCLC with co-mutations of 19.4%. We did not find any clinical or survival differences compared to the single-mutation population. However, the patients treated with targeted therapies as a first line had a shorter treatment period than those treated with a chemotherapy-based regimen, which, in addition, seems to obtain better OS and PFS.

Finally, we analysed the association between the previously validated inflammatory indexes [22,23,24,25] and outcomes after immunotherapy-based regimens for these oncogene-addicted-NSCLC patients. As is known from the literature [23], responses to immunotherapy seem to vary according to the NSCLC subtype, requiring potential tools for selecting patients who can respond in a better way. Among the indexes analysed, NLR showed some potential. This result suggests that these indexes may be tools for patient selection in these subpopulations. However, further, prospective evaluations in larger cohorts of patients should be performed in order to clarify their real role.

## 5. Conclusions

Our study focuses on subpopulations of oncogene-addicted NSCLCs with rare molecular alterations, as emerged in the retrospective, real-world-registry NERONE trial. We have shown that their incidence was lower than in the literature, with the exception of the ROS1 and erbB2 subgroups, with an incidence that was expected. The results in terms of the OS and PFS, although not statistically significant, show a tendency towards improved survival when specific targeted therapies are used from the beginning in the therapeutic strategy. Despite this, patients with co-mutations seem to receive an advantage from front-line chemotherapy-based regimens. Finally, the NLR score, a well-known inflammatory index, may have a relationship with the outcomes of immunotherapeutic-based strategies: in fact, when it is ≥4, it seems to be related to a worse OS in patients treated with immunotherapy alone or in combination with chemotherapy. This consideration seems to suggest that some patients with oncogene-addicted NSCLC may benefit from immunotherapy strategies. Given the limited number of patients and their molecular heterogeneity, these results are not conclusive and further prospective studies are warranted.

## Figures and Tables

**Table 1:** Baseline characteristics of patients with rare molecular alterations (*n* = 62).

	*n*	%
Sex		
M	27	(43.6)
F	35	(56.5)
Age at diagnosis (y)		
Median (IQ–IIIQ)	69.2 (65.0–76.2)	
Min–max	35.2–87.0	
<65	15	(24.2)
≥65	47	(75.8)
Smoking habit		
Never-smoker	23	(37.1)
Ex-smoker	28	(45.2)
Current smoker	11	(17.7)
ECOG PS		
0	19	(30.7)
1	30	(48.4)
2	10	(16.1)
3	2	(3.2)
4	1	(1.6)
BMI		
Median (IQ–IIIQ)	24 (22.0–28.0)	
Min–max	16–48	
<18.50	6	(9.7)
18.5–24.99	29	(46.8)
25.00–29.99	17	(27.4)
≥30.00	10	(16.1)
Histotype		
Adenocarcinoma	60	(96.8)
Squamous cell carcinoma	2	(3.2)
Type of treatment		
Target therapy	20	(32.3)
Chemo-immunotherapy	20	(32.3)
Immunotherapy	10	(16.1)
Chemotherapy	8	(12.9)
Clinical trial	4	(6.5)
PD-L1		
<1%	8	(13.1)
1–49%	35	(57.4)
≥50%	18	(29.5)
missing	1	
NLR		
Median (IQ–IIIQ)	4.0 (2.3–4.8)	
Min–max	0.6–25.2	
missing	13	
PLR		
Median (IQ–IIIQ)	194.4 (138.7–257.5)	
Min–max	23.1–779.6	
missing	12	
ALI		
Median (IQ–IIIQ)	24.6 (15.1–50.8)	
Min–max	3.5–56.7	
missing	41	
ALI		
<18	7	33.3
≥18	14	66.7
missing	41	
SII		
Median (IQ–IIIQ)	1095.2 (641.1–1842.1)	
Min–max	143.9–10,337.7	
missing	13	

Categorical variables are presented with absolute frequencies and percentages, while continuous variables are presented as median, first- and third-quartile, and minimum and maximum values. Percentages may not equal 100 due to rounding. IQ: first quartile; IIIQ: third quartile; BMI: body mass index; PD-L1: programmed death ligand 1; NLR: neutrophil-to-lymphocyte ratio; PLR: platelet-to-lymphocyte ratio; ALI: advanced lung cancer inflammation index; SII: systemic immune-inflammation index.

**Table 2:** Descriptive statistics of the demographic and clinical characteristics of the patients harbouring the most heavily represented rare molecular alterations.

	rROS1 (*n* = 15)		MET Ex.14 Skip (*n* = 11)		EGFR Ex.20 (*n* = 9)		ERBB2 (*n* = 8)	
	*n*	%	*n*	%	*n*	%	*n*	%
Sex								
M	7	(46.7)	6	(54.6)	1	(11.1)	2	(25.0)
F	8	(53.3)	5	(45.5)	8	(88.9)	6	(75.0)
Age at diagnosis (y)								
Median (IQ–IIIQ)	69.6 (55.3–76.2)		74.5 (69.0–78.5)		72.4 (63.2–76.3)		68.5 (63.3–69.2)	
Min–max	35.2–82.4		65.0–83.7		57.1–80.1		53.3–83.7	
<65	6	(40.0)	-		3	(33.3)	2	(25.0)
≥65	9	(60.0)	11	(100.0)	6	(66.7)	6	(75.0)
Smoking habit								
Never smoker	6	(40.0)	2	(18.2)	6	(66.7)	5	(62.5)
Ex-smoker	7	(46.7)	7	(63.6)	1	(11.1)	1	(12.5)
Current smoker	2	(13.3)	2	(18.2)	2	(22.2)	2	(25.0)
ECOG PS								
0	9	(60.0)	1	(9.1)	4	(44.4)	-	
1	4	(26.7)	8	(72.7)	4	(44.4)	5	(62.5)
2	1	(6.7)	2	(18.2)	1	(11.1)	2	(25.0)
3	-		-		-		1	(12.5)
4	1	(6.7)	-		-		-	
Histotype								
Adenocarcinoma	14	(93.3)	11	(100.0)	9	(100.0)	8	(100.0)
Squamous cell carcinoma	1	(6.7)	-		-		-	
Type of treatment								
Target therapy	13	(86.7)	1	(9.1)	-		-	
Chemo-immunotherapy	1	(6.7)	3	(27.3)	3	(33.3)	5	(52.5)
Immunotherapy	1	(6.7)	5	(45.5)	-		1	(12.5)
Chemotherapy	-		1	(9.1)	3	(33.3)	2	(25.0)
Clinical trial	-		1	(9.1)	3	(33.3)	-	
Presence of co-mutations								
No	10	(66.7)	10	(90.9)	9	(100.0)	6	(75.0)
Yes	5	(33.3)	1	(9.1)	-		2	(25.0)
PD-L1								
<1%	-		-		2	(22.2)	3	(37.5)
1–49%	10	(71.4)	5	(45.5)	4	(44.4)	5	(62.5)
≥50%	4	(28.6)	6	(54.6)	3	(33.3)	-	
Missing	1		-		-		-	
NLR								
Median (IQ–IIIQ)	2.4 (2.1–4.6)		4.2 (3.8–8.5)		2.2 (1.7–4.4)		4.4 (3.8–5.6)	
Min–max	0.6–15.4		2.1–12.7		0.8–6.7		1.9–9.4	
Missing	4		2		1		-	
PLR								
Median (IQ–IIIQ)	159.2 (108.6–234.2)		186.1 (151.7–240.9)		191.4 (130.3–235.4)		271.8 (170.4–321.7)	
Min–max	26.7–779.6		110.2–437.8		23.1–346.3		114.6–365.0	
Missing	4		2		1			
ALI								
Median (IQ–IIIQ)	43.5 (4.4–57.6)		14.9 (14.8–14.9)		55.4 (25.2–57.4)		17.3 (12.7–22.0)	
Min–max	4.2–58.0		14.8–14.9		25.2–57.4		12.7–22.0	
Missing	8		9		6		6	
ALI								
<18	2	28.6	2	(100.0)	-		1	(50.0)
≥18	5	71.4	-		3	(100.0)	1	(50.0)
Missing	8		9		6		6	
SII								
Median (IQ–IIIQ)	656.3 (519.4–1598.5)		1360.9 (1109.6–2724.2)		707.9 (483.3–1045.5)		1490.4 (1127.1–2056.7)	
Min–max	143.9–10,337.7		282.1–5130.7		226.1–1842.1		555.9–3004.6	
Missing	4		2		1		-	

Categorical variables are presented with absolute frequencies and percentages, while continuous variables are presented as median, first- and third-quartile, and minimum and maximum values. Percentages may not equal 100 due to rounding. IQ: first quartile; IIIQ: third quartile; BMI: body mass index; PD-L1: programmed death ligand 1; NLR: neutrophil-to-lymphocyte ratio; PLR: platelet-to-lymphocyte ratio; ALI: advanced lung cancer inflammation index; SII: systemic immune-inflammation index; -: no result present.

**Table 3:** Results from univariate analyses using the Cox model of the association between the inflammatory indexes and the overall survival (OS) and progression-free survival (PFS).

	OS			PFS		
	HR	(95% CI)	*p*-Value	HR	(95% CI)	*p*-Value
NLR						
<4.0	1.0			1.0		
≥4.0	2.16	(0.94–4.93)	0.069	1.42	(0.71–2.84)	0.327
Log-transformed NLR	1.37	(0.73–2.56)	0.324	1.17	(0.71–1.94)	0.539
PLR						
<194.4	1.0			1.0		
≥194.4	1.45	(0.65–3.25)	0.364	1.18	(0.59–2.37)	0.635
Log-transformed PLR	0.96	(0.52–1.76)	0.890	1.01	(0.62–1.64)	0.968
SII						
<1095.2	1.0			1.0		
≥1095.2	1.92	(0.84–4.38)	0.123	1.28	(0.63–2.57)	0.483
Log-transformed SII	1.17	(0.72–1.90)	0.532	1.06	(0.72–1.58)	0. 774
ALI						
<18	1.0			1.0		
≥18	0.75	(0.17–3.35)	0.705	0.46	(0.15–1.45)	0.188
Log-transformed ALI	0.89	(0.37–2.13)	0.797	0.83	(0.45–1.57)	0.581

HR: hazard ratio; CI: confidence interval; NLR: neutrophil-to-lymphocyte ratio; PLR: platelet-to-lymphocyte ratio; ALI: advanced lung cancer inflammation index; SII: systemic immune-inflammation index.

## Data Availability

The data that support the findings will be available in www.zenodo.org at https://doi.org/10.5281/zenodo.11164367 following receipt of approval from Privacy Guarantor.
